# Cucurbitane Glycosides Derived from Mogroside II_E_: Structure-Taste Relationships, Antioxidant Activity, and Acute Toxicity

**DOI:** 10.3390/molecules190812676

**Published:** 2014-08-20

**Authors:** Lei Wang, Ziming Yang, Fenglai Lu, Jinglei Liu, Yunfei Song, Dianpeng Li

**Affiliations:** 1 Guangxi Key Laboratory of Functional Phytochemicals Research and Utilization, Institute of Botany, Guangxi Zhuang Autonomous Region and Chinese Academy of Sciences, Guilin 541006, China; 2 Guilin Layn Natural Ingredients Corp., Guilin 541199, China

**Keywords:** mogroside, sweetener, cucurbitane glycosides, cyclodextrin glucanotransferase

## Abstract

Mogroside IIE is a bitter triterpenoid saponin which is the main component of unripe Luo Han Guo fruit and a precursor of the commercially available sweetener mogroside V. In this study, we developed an enzymatic glycosyl transfer method, by which bitter mogroside IIE could be converted into a sweet triterpenoid saponin mixture. The reactant concentration, temperature, pH and buffer system were studied. New saponins with the α-glucose group were isolated from the resulting mixtures, and the structures of three components of the extract were determined. The structure-taste relationships of these derivatives were also studied together with those of the natural mogrosides. The number and stereoconfiguration of glucose groups present in the mogroside molecules were found to be the main factor to determine the sweet or bitter taste of a compound. The antioxidant and food safety properties were initially evaluated by their radical scavenging ability and via 7 day mice survival tests, respectively. The results showed that the sweet triterpenoid saponin mixture has the same favorable physiological and safety characteristics as the natural mogrosides.

## 1. Introduction

Luo Han Guo, or *Siraitia grosvenori* Swingle, a traditional Chinese medicine and edible fruit, is a member of the gourd family. Extracts from ripe Luo Han Guo fruit are intensely sweet. Additionally, because of the low calorie content and other beneficial health effects, the extracts are excellent commercial sugar substitutes [1,2]. The taste of Luo Han Guo fruits and their extracts is due to a mixture of cucurbitane-type triterpene glycosides, the mogrosides, with mogroside V being the major component of the ripe fruit. The relative sweetness of mogroside V is over 300 times higher than that of sucrose [3,4].

Mogrosides are known to function as antioxidants, anti-carcinogens, and anti-inflammatory substances. Numerous papers have reported that when administered to diabetic mice mogrosides may prevent diabetic complications via their strong antioxidant properties [5,6,7]. Related studies have reported that mogrosides exhibit potent inhibitory effects on early Epstein-Barr virus antigens and the carcinogenesis of mouse skin tumors [8,9,10,11]. Di’s research assessed the anti-inflammatory properties of mogrosides in both murine macrophage RAW264.7 cells and a murine ear edema model, and showed that the anticancer and antidiabetic effects of mogrosides may result, in part, from their anti-inflammatory activity [12].

The biogenetic precursor of mogroside V, mogroside II_E_, has a bitter taste and is present mainly in immature fruit. Our previously reported research showed that mogroside II_E_ is the predominant saponin component in fruits that are less than 45 days old [13]. The bitter compound slowly disappears in the fruits up to an age of 70 days, and during the same period the presence of mogroside V gradually builds up. Each year, during the late harvest season, considerable quantities of bitter fruit, which did not ripen due to variable weather conditions, are discarded on the branches. Although attempts were made over 20 years ago to improve the cultivation and breeding methods in order to reduce the impact of growth stagnation, effective methods for prevention of the problem of unripe fruits entering the production pipeline are still in high demand [14]. Moreover, as the worldwide demand for mogroside V gradually increases, the potential value of this bitter fruit waste stream becomes more prominent.

In careful studies of the relationship between the structure and the taste of mogrosides, we found that the number of glucose units included in the mogroside molecule determines the taste [4,15,16,17]. If the number of glucose units in the mogrosides in Luo Han Guo fruit is four or greater, the compound is sweet, while less than four glucose units results in a bitter taste. With this basic understanding, it may be possible to affect a glycosylation reaction *in vitro* to extend the sugar chains of the bitter mogrosides in order to convert them into sweet substances.

## 2. Results and Discussion

In the present study, sweet mogrosides were produce by tranfering glucose residues to the diglycoside mogroside II_E_, by catalysis with cyclodextrin glucanotransferase (CGTase). Three novel structural mogrosides were isolated from the reaction mixture and structurally characterized, and the corresponding structure-taste relationships evaluated. The antioxidant capability and acute toxicity assessment of these mogroside II_E_ derivatives were also examined.

### 2.1. Transglycosylation of Mogroside II_E_ with CGTases

CGTase is a versatile enzyme capable of catalyzing hydrolysis and transglycosylation reactions. It is mainly used to produce cyclodextrins (CDs) from starch [18,19,20]. CGTase also exhibits a strong ability for catalyzing intermolecular transglycosylation, by which stevioside and rebaudioside A have been modified to improve their taste and water solubility [21,22].

**Figure 1:**
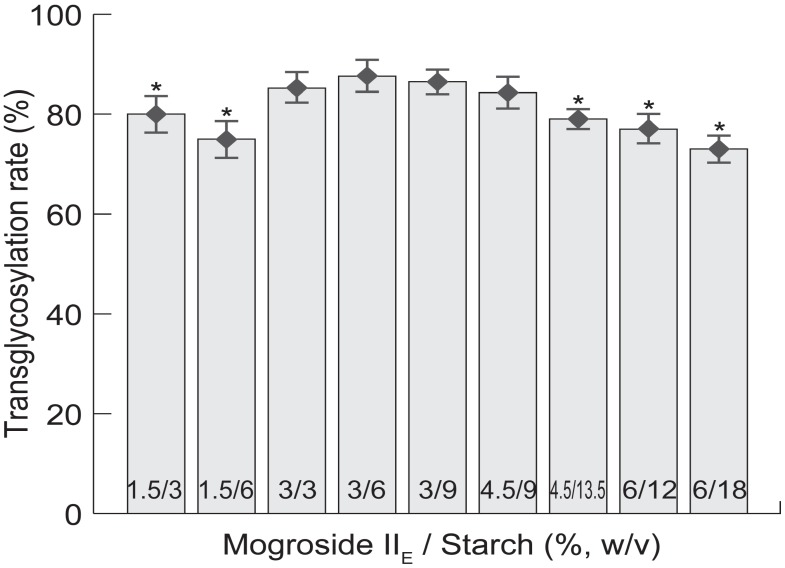
Transglycosylation rate affected by mogroside II_E_/starch concentration ratios.

Reaction conditions were optimized on the basis of reactant concentration ratio, temperature, reaction time, and pH. Figure 1 shows how the transglycosylation rate is affected by mogroside II_E_/starch concentration ratios under conditions of transglycosylation at 55 °C, pH 6, and in the presence of CGTase (30 U/g mogroside II_E_) for 8 h. when the concentration of mogroside II_E_ is 3%, the transglycosylation rate is not greatly influenced by starch concentration. Moreover, when the concentration ratio of mogroside II_E_ and starch was 1:2, the transglycosylation rate was significantly high. An increased proportion of starch did not enhance the reaction rate. This finding indicates that the presence of too much starch may reduce intermolecular transglycosylation and result in a relatively low yield. Therefore, the optimal concentrations of mogroside II_E_ and starch are 3% and 6%, respectively.

* Significantly different (*p* < 0.05) from the 3/6 sample.

**Figure 2:**
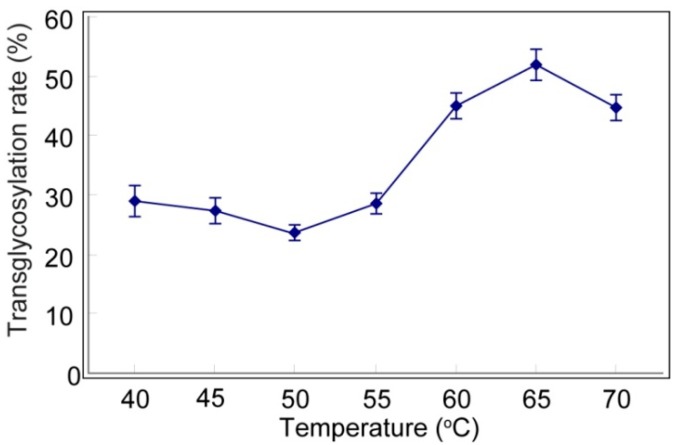
Transglycosylation rate affected by temperature.

A series of temperature tests were then performed at pH 6 and mogroside II_E_/starch = 3/6, in the presence of CGTase (35 U/g mogroside II_E_) for 2 h (Figure 2). From this picture, the transglycosylation rate increased with an increase in temperature and reached a maximum rate at 65 °C. Reactions conducted at 60–65 °C would not only ensure a robust reaction rate, but also ensure the stability of CGTases, thus, this temperature range represents the optimum reaction temperature.

**Figure 3:**
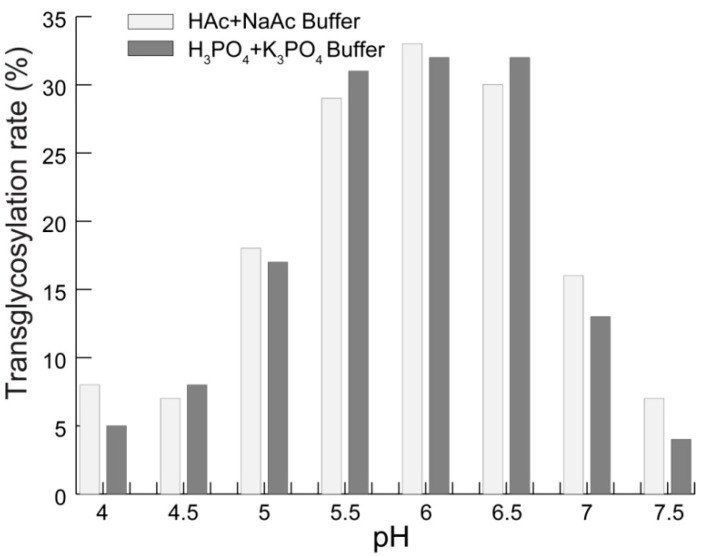
Transglycosylation rate affected by pH.

Enzymatic reactions are strongly influenced by pH and the presence of ions in the reaction medium. Therefore, two commonly used buffers (HAc-NaAc and H_3_PO_4_-K_3_PO_4_) were introduced to test their effect on the reaction conditions. Figure 3 shows that the transglycosylation rate is obviously enhanced between pH 5.5–6.5 in the presence of CGTase (40 U/g mogroside II_E_), 60 °C, and mogroside II_E_/starch = 3/6 for 1 h. Moreover, there was no significant difference between the two buffers in the tested pH range.

**Figure 4:**
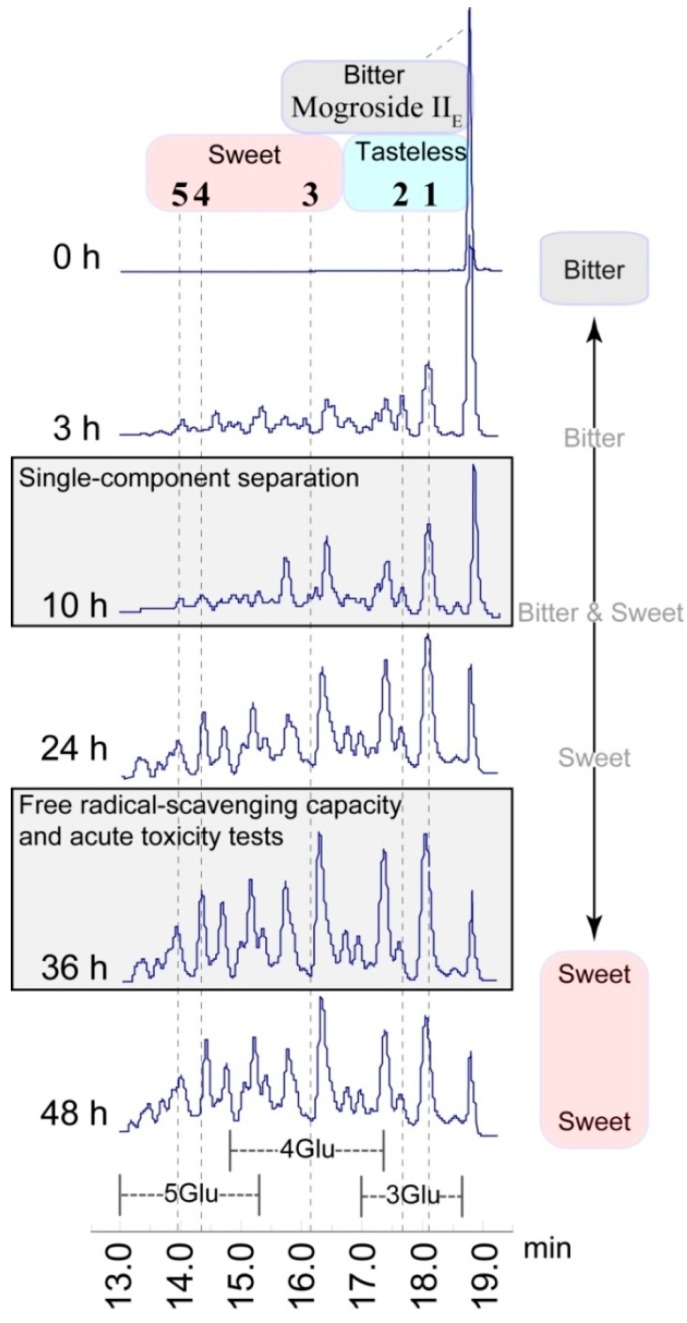
Product changes detected by HPLC-MS.

With the results of these experiments, we found that the optimal reaction conditions are 3% mogroside II_E_ and 6% starch concentration, temperature between 60 and 65 °C, and a pH range of 5.5–6.5. As previously noted, differing numbers of sugar residues incorporated into the structure of mogroside result in a completely different taste experience. Therefore, a long-term experiment was carried out using the HPLC-MS method to study product changes with time. As shown in Figure 4, along with the rapid disappearance of mogroside II_E_, a complex mixture was produced. The bulk of the product was comprised of saponins containing 3–5 sugar units, together with very small quantities of saponins containing six glucose units appearing around 13 min in the HPLC chromatogram. In particular, the loss of mogroside II_E_ slowed down after 24 h, and less than 4% remained which could not be completely reacted. It is likely due to the lower limit of binding concentration having been achieved for this enzyme reaction. During the 24–48 h time period, the relative content of saponins containing three sugar residues decreased and a relative content of saponins containing 4–5 sugar residues increased. This phenomenon suggested that saponins containing three sugar residues had become the main acceptor of intermolecular transglycosylation. Qualitative taste testing showed that the bitter taste of mogroside II_E_ had disappeared after 24 h, while the sweetness did not increase further until after 36 h. Therefore, an optimal reaction time was 24–36 h.

### 2.2. Structure-Taste Relationships

**Figure 5:**
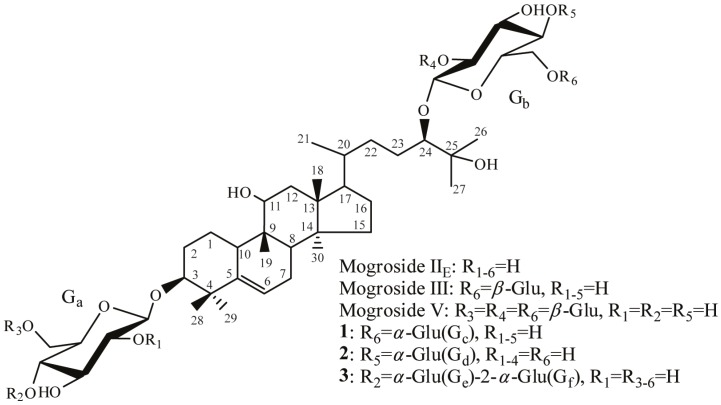
Structure of compounds **1**–**3** and other mentioned natural mogrosides.

Based on the HPLC chromatograms, and as expected, the composition of the product was highly complex. Using preparative HPLC, five components were purified. Their chemical structures were identified by 1D and 2D-NMR together with ESI-MS. The structure of compounds **1**–**3** are shown in Figure 5.

CGTase is an *α*-1,4-glucosidase used to synthesize cyclodextrin. However, its regioselectivity is not prominent in this saponin transglycosylation reaction. 1,6- and 1,2-linkage products were identified and all newly generated glycosidic bonds were shown to be in an *α*-orientation. In addition, all transglycosylation reactions occurred at the hydroxyl groups of the glucose moiety rather than the aglycone part of mogroside II_E_. The glucose group is a more suitable receptor for the transglycosylation reaction.

Compound **1** is a trisaccharide saponin with a newly formed *α*-glycosidic bond at the 6-methylene of the 24-glucose of mogroside II_E_. HMBC-NMR experiments showed a cross peak correlation between C-1 (δ_C_ 99.44) of the new glucose (G_c_) and the H-6 (δ_H_ 4.20, 4.51) of the 24-glucose (G_b_) moiety. The NOE difference spectrum experiment demonstrated that when the glucose (G_c_) H-1 signal (δ_H_ 5.44) was saturated, two NOE difference signals were produced at δ 4.20 and δ 4.51 ppm (H-6 of G_b_). Analysis of the ^13^C-DEPT, ^1^H-^1^H COSY, ROESY, HSQC, and ESI-MS spectra of **1** were consistent with the proposed structure.

Compound **2** is an isomer of compound **1** with a newly formed *α*-glucose attached to C-4 of the C-24 linked glucose of mogroside II_E_. The presence of a new glycosidic bond was supported by the observation of a cross peak correlation in the HSQC spectrum (δ_C_ 102.80 ppm and δ_H_ 5.88 ppm). The H-1 (G_d_) and C-4 (G_b_) cross-peak in the HMBC spectrum and the Overhauser effect between H-1 (G_d_) and H-4 (G_b_) in the NOE difference spectrum showed that a new glycosidic bond was connected to the 4-methine of 24-Glu. This evidence, coupled with the interpreted results of the ^13^C-DEPT, ^1^H-^1^H COSY, ROESY, HSQC, and ESI-MS spectra, were consistent with structure **2**.

Compound **3** contains four glucose groups with an *α*-Glu-1→2-*α*-Glu structure at the 4-methine of the 3-glucose. The ^13^C- and ^1^H-NMR spectra of **3** showed two new glycosidic bonds, and correlation peaks between their ^1^H signals with the same ^13^C signal were found in HMBC spectrum, which indicated that the two glucose units were connected to each other by a 1→2 linkage glycosidic bond. Furthermore, an H-1 (G_e_) and C-4 (G_a_) correlation cross peak could be observed in the HMBC spectrum, and showed that the G_a_ glucose and the G_e_ glucose were connected through a 1→4 linkage. The NOE difference spectra showed separately that when the H-1 of each of the two new glucose units was saturated, the Overhauser effects appeared at the point of connection which also corresponded to the correlation results from the HMBC spectrum. The results of other NMR and ESI-MS experiments were also consistent with structure **3**.

Due to severe overlap of the glucosyl signals in the NMR spectra, the exact molecular structure of compounds **4** and **5** could not be determined. Based on known NMR and MS information, it can be stated that they are all pentasaccharide saponins containing three new *α*-glycosidic bonds. More detailed NMR data of compounds **1**–**5** is summarized in the Supplementary Information.

The synthetic mogroside II_E_ derivatives **3**–**5** exhibit a sweet taste. This phenomenon is consistent with the existing knowledge that all natural mogrosides containing four or more glucose units are sweet. However the intensity (degree) of sweetness exhibited by **3**–**5** is much reduced relative to that of mogroside V or mogroside IV, taken at the same concentration. Moreover, the trisaccharide saponins, **1** and **2**, have a subtle and unique taste that is neither sweet nor bitter, but is significantly different from the very bitter taste of the natural triglycoside, mogroside III. While 4% or less of mogroside II_E_ does not react in the CGTase catalyzed transglycosylation reaction, the residual content of this diglycoside does not affect the taste of the final product. The triterpenoid saponin mixture obtained after enzymatic transglycosidation is slightly sweet, with a hint of a special side taste due to compounds **1** and **2**.

The following findings of a relationship between structure and taste should be noted. The difference between compound **1** and mogroside III is the stereo configuration of the glycosidic bond at the R6 position, shown in Figure 5. While mogroside III is very bitter, compound **1** is nearly tasteless. The sweetness of compounds **4** and **5** containing *α*-glycosidic bonds is much reduced compared to that of mogroside V. Moreover, compounds **1** and **2** have almost the same taste. To summarize the above findings and literature, three conclusions can be drawn: first, the number of sugar residues appears to be the decisive factor affecting mogroside taste. Second, the *β*-glycosidic bond enhances the sweet taste experience, while the *α*-glycosidic bond does not have this ability. Third, the point of interglycosidic linkage of the glucose unit has only a minor effect on the taste.

### 2.3. Free Radical Scavenging Capacity

As mentioned earlier, natural mogrosides exhibit good health benefits when used as sweeteners. This comes mainly from their antioxidant activities [5,6,7]. It was therefore important to establish whether these new artificial mogrosides exhibited the same characteristics. To test their antioxidant ability, free radical scavenging tests are performed. These measurements include testing the scavenging effects on hydroxyl radicals, superoxide radicals, and DPPH. Since the product is complex and separating any single component is difficult, the following tests were conducted on the mixed saponin product (MSP). Results of free radical scavenging capacity are shown in Table 1.

**Table 1:** IC_50_ values of free radical scavenging tests ^a^.

Tests	MSP	Mogroside V
Hydroxyl Radicals	0.202	0.139
Superoxide Radicals	1.260	0.551
DPPH	0.954	0.433

^a^ Calculated by nonlinear fitting under condition of R^2^ > 0.99.

The hydroxyl-radical scavenging activities of MSP and mogroside V were evaluated by their ability to prevent damage to d-deoxyribose using the 2-thiobarbituric acid methods, and the scavenging effects were measured as IC_50_ values. Several sample concentrations were measured, and the dose/scavenging rate relationship was recorded. The IC_50_ values were calculated by nonlinear fitting to be 0.202 mg/mL and 0.139 mg/mL for MSP and mogroside V, respectively.

Based on the pyrogallol method, the ability of MSP and mogroside V to scavenge superoxide free radicals was evaluated. The IC_50_ values for MSP and mogroside V were 1.260 mg/mL and 0.551 mg/mL, respectively. Using the DPPH method, another widely used free radical scavenging evaluation was also carried out, and the IC_50_ values were 0.954 mg/mL and 0.433 mg/mL for MSP and mogroside V, respectively.

The IC_50_ values show that the free radical-scavenging capacity of MSP was slightly weaker than that of mogroside V. This is mainly due to the fact that MSP is a mixture in which some components may have only weak free radical-scavenging activity. Nevertheless, these data indicate that MSP maintains the free radical-scavenging properties of mogrosides, despite the structural modifications. Notably, further tests are needed to determine whether MSP inherited all of the health effects of the mogrosides.

### 2.4. Acute Toxicity Tests

During a 7-day experimental period, no death or anomalies in general appearance were observed in the treated groups. No tissue lesions were found in any of the tested mice. On the basis of serum biochemistry and organ weight data after the acute toxicity tests (shown in Table 2), it was determined that there was no significant difference in this regard between the control and treated groups. The four important serum biochemical parameters—ALT, AST, CRE, and BUN—were all in the normal range relative to the control group. These results indicated that the liver and renal functions of tested mice were not injured to the 15 mg/g body weight dose of MSP. We speculate that similar to mogroside V [23], the relatively low absorption rate of saponins would ensure high tolerance of MSP.

**Table 2:** Serum biochemical and organ weight data after acute toxicity tests.

Parameters	Treated Group		Control Group	
	Male	Female	Male	Female
ALT (U/L)	30.1 ± 4.9	25.6 ± 3.1	27.3 ± 3.6	26.0 ± 2.7
AST (U/L)	55.8 ± 7.2	59.4 ± 5.5	52.3 ± 6.1	56.1 ± 6.5
CRE (mmol/L)	42.7 ± 10.4	37.9 ± 8.3	43.8 ± 9.9	39.6 ± 10.0
BUN (mmol/L)	6.5 ± 1.2	6.8 ± 1.5	6.3 ± 1.8	6.6 ± 1.7
Liver Weight (g)	2.92 ± 0.087	1.55 ± 0.051	2.93 ± 0.107	1.57 ± 0.079
Kidney Weight (g)	0.60 ± 0.022	0.34 ± 0.032	0.58 ± 0.048	0.34 ± 0.035
Spleen Weight (g)	0.09 ± 0.004	0.10 ± 0.003	0.10 ± 0.006	0.10± 0.004
Body Weight at the Beginning (g)	29.5 ± 2.7	24.1 ± 2.6	29.5 ± 2.6	24.0 ± 2.8
Body Weight at the End (g)	32.7 ± 5.1	27.6 ± 6.3	32.5 ± 6.0	28.0 ± 5.9

## 3. Experimental Section

### 3.1. Chemicals and Materials

Mogroside II_E_ was prepared from fresh 20–30 day-old Luo Han Guo fruits. The fruits were crushed and boiled in water (three times) at 100 °C for 1 h. After filtration, the combined filtrate was subjected to D101 column chromatography using H_2_O/EtOH (1:0–2:8), the part of eluent from 50%–80% EtOH was collected. This fraction was further separated by ODS column chromatography, and mogroside II_E_ was eluted with 45% EtOH. Cyclodextrin glucanotransferase (EC. 2.4.1.19, CGTase) was purchased from Amano Enzyme Inc. (Nagoya, Japan). Other chemicals were reagent grade and used as received without further purification.

### 3.2. General Procedures

All NMR spectra were carried out at room temperature on a 500 MHz (^1^H-NMR) and 125 MHz (^13^C-NMR) Bruker instrument (Bruker, Billerica, MA, USA). Chemical shifts (δ) were expressed relative to the chemical shift of the NMR solvent. Coupling constants (*J*) values were expressed in hertz (Hz).

HPLC for measurement of mogrosides was performed on an Agilent 1200 HPLC (Santa Clara, CA, USA) using the following conditions: Column, YMC-pack ODS-AQ (4.6 × 250 mm, YMC Co., Kyoto, Japan); Solvent, 13.5%–35% (v/v) acetonitrile in 0–25 min; Flow rate, 0.8 mL/min; Temperature, 30 °C; Detection wavelength, 209 nm.

LC-MS was performed on an Agilent 1100 LC/MSD Trap instrument. The chromatographic conditions were same as above HPLC measurement. ESI-MS detection was performed in negative ion mode with mass acquisition between 150 and 2000 Daltons. Nitrogen was used as drying and nebulizer gas at 45 psi, 350 °C and at a flow rate of 8 L/min.Elemental analysis was performed on a Vario EL III instrument (Elementar Analysensysteme GmbH, Hanau, Germany). Carbon and hydrogen were determined three times and reported as an average value.

### 3.3. Transglycosylation Ratio

The transglycosylation reaction of mogroside II_E_ by CGTases was detected by peak area reduction of mogroside II_E_ in the HPLC spectra. Transglycosylation ratios (%) were calculated from the peak area ratio of mogroside II_E_ and its transglycosylation products according to HPLC spectra under the measurement conditions as described above.

### 3.4. Hydroxyl Radical-Scavenging Assay

To determine the rate of the hydroxyl radical reaction with antioxidants, the deoxyribose method was performed with minor modifications [24]. The reaction mixture (2.0 mL volume) contained d-deoxyribose (0.4 mL × 30 mmol/L), ascorbic acid (0.2 mL × 0.1 mmol/L), FeCl_3_ (0.2 mL × 0.1 mmol/L), EDTA (0.2 mL × 0.103 mmol/L), H_2_O_2_ (0.2 mL × 1 mmol/L), KH_2_PO_4_-K_3_PO_4_ buffer (pH 7.4, 0.6 mL × 20 mmol/L), and 0.2 mL of test samples at different concentrations. Solutions of FeCl_3_ and ascorbic acid were freshly prepared before use. The reaction mixture was incubated at 37 °C for 1 h, and then 2 mL of a 0.5% 2-thiobarbituric acid (w/v) and 12% (v/v) HCl mixture was added. The final solution was then heated in a boiling water bath for another 15 min. After cooling to room temperature, the absorbance of the resulting solution was measured at 532 nm. The scavenging rate (%) was calculated by Equation (1), as follows:

Scavenging rate (%) = [A_i_ − (A − A_0_)]/A_i_ × 100 (1)

where A_i_ is the absorbance of the solution without the sample, A_0_ is the absorbance of the solution without d-deoxyribose, and A is the absorbance in the presence of test samples.

### 3.5. Superoxide Radical Scavenging Assay

Measurement of superoxide anion-scavenging activity was based on the reported method with slight modifications [25]. Tris-HCl buffer (pH 8.2, 4.5 mL × 50 mmol/L) and 1.0 mL of tested sample in various concentrations were mixed in tubes with lids. After incubation at 25 °C for 20 min, a solution of pyrogallol (0.4 mL × 25 mmol/L, preheated at 25 °C) was added to the mixture. After waiting four minutes, the reaction was terminated with addition of 0.1 mL of 6 mol/L HCl. The absorbance of the resulting solution was measured at 325 nm and the scavenging rate was calculated using Equation (2):

Scavenging rate (%) = (A_0_ − A)/A_0_ × 100 (2)

where A_0_ is the absorbance without the sample and A is the absorbance with the sample.

### 3.6. DPPH Radicals Scavenging Assay

The free radical scavenging capacity was analyzed using the DPPH test according to the reference with some modifications [26]. Samples (3.0 mL) at various concentrations (0.01 to 5.0 mg/mL) were added to a DPPH (1 mL × 0.1 mmol/L) methanol solution. The absorbance at 517 nm was measured after the solution was kept at room temperature for 30 min. The DPPH radical scavenging rate was calculated using Equation (2).

### 3.7. Acute Toxicity Tests

Kunming mice were grouped and housed in a controlled environment (temperature, 25 ± 1 °C; humidity, 50% ± 10%; 12 h light/12 h dark cycle) in the institutional animal facility with *ad libitum* access to food and water. The animals were acclimatized for a week before use. For subsequent acute toxicity experiments 8 male and 8 female mice were used. A solution of the test sample was administered once to mice at a 15 mg/g body weight dose by gavage [27]. To a control group was administered the same volume of water. Mice were bred for a week, during which their general appearance, body weight, food and water consumption were monitored and recorded. Blood was obtained from the eyeballs of the mice, after which they were sacrificed. Serum biochemical parameters, organ weight, and histopathological findings for the tissue specimens were analyzed and compared with the control and treated groups. Biochemical parameters were estimated using commercially available kits (Shanghai Yajimeilian Biological Technology Co., Ltd., Shanghai, China) for alanine aminotransferase (ALT), aspartate aminotransferase (AST), creatinine (CRE), and blood urea nitrogen (BUN).

### 3.8. Preparation of Compounds

Mogroside II_E_ (3%) and 6% starch were dissolved in a 50 mL pH 5.5–6.5 H_3_PO_4_-K_3_PO_4_ buffer, and then 40 U/g CGTase (by mogroside II_E_ weight) was added. Under slow stirring the mixture was heated to 60–65 °C for a certain time. The reaction time for free radical-scavenging capacity and acute toxicity tests was 36 h, while for single-component separation was 10 h. After boiling for 5 min to terminate the reaction, the solution was cooled to room temperature and filtered. The final reaction filtrate was layered on a column packed with YMC GEL ODS-A. A step gradient elution was applied, and the 20%–50% EtOH fraction was collected and dried. The final white powder was a mixture of triterpene glycosides.

### 3.9. Taste Analysis

Compounds **1**–**5**, MSP, mogroside V, mogroside II_E_, and mogroside III were dissolved in distilled water to make 0.5% solutions, respectively. Five volunteers tasted each solution and wrote down their taste experience as “sweet, slightly sweet, tasteless, slightly bitter, or bitter”, and make an order of each sample in accordance with the sweet intensity. In the HPLC-MS monitored long-term experiment, 3 mL of reaction solution was taken out at certain sampling time. Then the sample solution was diluted to 10 mL, boiled 2 min, and sealed in the refrigerator. All samples were tested together by three volunteers following the method described above.

### 3.10. Identification and Characterization

Three compounds were separated and identified by MS, elemental analysis and NMR (^1^H-, ^13^C-, NOE difference spectra, DEPT, HSQC, HMBC, ROESY, and TOCSY).

*3-O-β-d-Glucopyranosyloxomogrol 24-O-β-d-glucopyranosyl-(1→6)-α-d-glucopyranoside* (**1**): ESI-MS, *m/z* 962 [M−1]^−^; ^1^H-NMR (pyridine-d_5_ with a drop of D_2_O): δ 0.81 (3H, s), 0.89 (3H, d, *J* = 6.5), 0.91 (3H, s), 1.14 (3H, s), 1.20 (2H, m), 1.27 (3H, s), 1.30 (3H, s), 1.36 (1H, m), 1.39 (3H, s), 1.47 (1H, m), 1.49 (1H, m), 1.55 (3H, s), 1.64 (3H, m), 1.70 (2H, m), 1.81 (1H, m), 1.90 (1H, m), 1.99 (2H, m), 2.05 (2H, m), 2.27 (1H, m), 2.42 (1H, m), 2.76 (1H, m), 2.91 (1H, m), 3.67 (1H, m), 3.72 (1H, m), 3.88–4.01 (3H, m), 4.07 (2H, m), 4.13–4.25 (5H, m), 4.37 (4H, m), 4.45 (1H, s), 4.51 (3H, m), 4.69 (1H, t, *J =* 9), 4.86 (1H, d, *J =* 7.5), 4.88 (1H, d, *J =* 10.5), 5.44 (1H, d, *J =* 3.0), 5.46 (1H, d, *J =* 5.5); ^13^C-NMR (pyridine-d_5_) δ 16.78, 18.56, 19.06, 24.33, 25.12, 25.99, 26.09, 26.51, 26.62, 27.47, 28.04, 29.30, 29.57, 32.99, 34.31, 36.19, 36.60, 39.89, 40.86, 42.12, 43.27, 47.15, 49.46, 50.70, 62.62, 62.81, 67.63, 71.58, 72.12, 72.18, 72.31, 73.90, 74.12, 74.84, 75.25, 77.53, 77.57, 77.85, 78.33, 78.44, 87.62, 91.94, 99.44, 106.03, 107.07, 118.24, 144.00; Anal. Calcd for C_48_H_82_O_19_·3H_2_O: C, 56.68; H, 8.72; O, 34.60. Found: C, 56.43; H, 8.63.

*3-O-β-d-Glucopyranosyloxomogrol 24-O-β-d-glucopyranosyl-(1→4)-α-d-glucopyranoside* (**2**): ESI-MS, *m/z* 962 [M−1]^−^; ^1^H-NMR (pyridine-d_5_ with a drop of D_2_O) δ 0.82 (3H, s), 0.89 (3H, s), 0.95 (3H, d, *J =* 6), 1.08 (2H, m), 1.12 (2H, m), 1.14 (3H, s), 1.31 (3H, s), 1.36 (3H, s), 1.38 (1H, m), 1.41 (2H, s), 1.44 (1H, m), 1.52 (2H, m), 1.54 (3H, s), 1.67 (4H, m), 1.78 (1H, m), 1.88 (1H, m), 1.99 (1H, m), 2.06 (2H, m), 2.27 (1H, m), 2.42 (1H, m), 2.77 (1H, m), 2.90 (1H, m), 3.66 (1H, m), 3.82 (1H, m), 3.92 (4H, m), 4.00 (1H, m), 4.14-4.21 (6H, m), 4.29 (4H, m), 4.36 (1H, m), 4.47–4.55 (3H, m), 4.88 (1H, d, *J =* 10.5), 4.90 (1H, d, *J =* 8.0), 5.47 (1H, s), 5.88 (1H, d, *J =* 2.8); ^13^C-NMR (pyridine-d_5_) δ 16.79, 18.60, 19.09, 24.33, 25.15, 26.00, 26.09, 26.52, 26.61, 27.47, 28.07, 29.20, 29.30, 33.26, 34.33, 36.31, 36.61, 39.89, 40.85, 42.12, 43.27, 47.15, 49.48, 50.67, 61.59, 62.46, 62.76, 71.58, 71.75, 71.87, 74.13, 74.61, 75.03, 75.23, 76.59, 77.42, 77.58, 77.85, 78.26, 78.36, 80.93, 87.63, 90.33, 102.80, 105.36, 107.05, 118.24, 144.01; Anal. Calcd for C_48_H_82_O_19_·6H_2_O: C, 53.82; H, 8.84; O, 37.34. Found: C, 54.01; H, 8.79.

*3-O-β-d-Glucopyranosyl-(1→4)-(α-d-glucopyranoside-(1→2)-α-d-glucopyranosyl)-oxomogrol 24-O-β-d-glucopyranoside* (**3**): ESI-MS, *m/z* 1124 [M−1]^−^; ^1^H-NMR (pyridine-d_5_ with a drop of D_2_O) δ 0.82 (3H, s), 0.89 (3H, s), 0.95 (3H, d, *J =* 6.5), 1.05 (1H, m), 1.12 (3H, s), 1.18 (1H, m), 1.25 (1H, m), 1.30 (3H, s), 1.38 (4H, s), 1.44 (3H, m), 1.53 (4H, m), 1.62 (1H, m), 1.68 (3H, m),1.81 (2H, m), 1.90 (1H, m), 2.06 (4H, m), 2.28 (1H, m), 2.35 (1H, m), 2.76 (1H, m), 2.89 (1H, m), 3.64 (1H, m), 3.80 (1H, m), 3.86 (1H, m), 3.92 (2H, m), 4.00–4.08 (3H, m), 4.16–4.26 (7H, m), 4.28–4.34 (3H, m), 4.39 (4H, m), 4.45-4.60 (4H, m), 4.66 (1H, m), 4.85 (1H, d, *J =* 7.69), 4.98 (1H, d, *J =* 8.2), 5.47 (1H, s), 5.79 (1H, d, *J =* 3.5), 5.90 (1H, d, *J =* 3.5); ^13^C-NMR (pyridine-d_5_) δ 16.80, 18.59, 19.06, 24.33, 25.08, 25.98, 26.06, 26.47, 26.73, 27.42, 28.06, 29.20, 29.30, 33.21, 34.31, 36.22, 36.59, 39.88, 40.87, 42.08, 43.26, 47.15, 49.46, 50.69, 61.65, 61.82, 62.46, 62.48, 71.51, 71.63, 71.67, 71.81, 73.24, 73.82, 74.27, 74.76, 74.82, 74.99, 75.13, 75.20, 76.38, 77.56, 77.63, 78.27, 78.34, 81.35, 87.71, 90.48, 102.65, 102.94, 105.66, 106.90, 118.24, 143.96; Anal. Calcd for C_54_H_92_O_24_·5H_2_O: C, 53.36; H, 8.46; O, 38.18. Found: C, 53.10; H, 8.55.

## 4. Conclusions

In summary, the technology to convert otherwise useless mogroside II_E_ into a product exhibiting a sweet taste using CGTase is viable. Although the structurally similar synthetic mogroside II_E_ derivatives are not sweeter than natural mogroside V, they do inherit comparable physiological and safety characteristics of the natural mogrosides, while exhibiting a somewhat reduced level of sweetness. As long as major breakthroughs in Luo Han Guo agricultural technology cannot be achieved, the methodology described here could represent a viable approach of converting bitter fruit material that is currently discarded as waste into useful products by means of enzymatic conversion. Moreover, if a glucanotransferase capable of forming *β*-glycoside linkages can be identified, the product so formed with this novel enzyme would likely compare favourably with mogroside V.

## Supplementary Materials

Supplementary materials can be accessed at: http://www.mdpi.com/1420-3049/19/8/12676/s1.

## Supplementary Files

Supplementary File 1
