# Supplementary material for: Cucurbitane Glycosides Derived from Mogroside IIE: Structure-Taste Relationships, Antioxidant Activity, and Acute Toxicity
Source: Molecules. 2014 Aug 20;19(8):12676–89. doi: 10.3390/molecules190812676 (PMC6271920; doi:10.3390/molecules190812676)
Supplement: Supplementary File 1 [file molecules-19-12676-s001.pdf]

Supplementary Materials

Table S1. NMR data of sugar parts of compound **1–3**.

No.	**1**				**2**				**3**			
	δ_C	δ_H	HMBC (C to H)	NOE [a] (H to H)	δ_C	δ_H	HMBC (C to H)	NOE [a] (H to H)	δ_C	δ_H	HMBC (C to H)	NOE [a] (H to H)
G_a1	107.07	4.88 (d, J = 10.5)	3, G_a2	3, G_a2, G_a3	107.05	4.90 (d, J = 8.0)	3, G_a2	3, G_a2, G_a3	106.9	4.85 (d, J = 7.69)	3, G_a2	3, G_a2, G_a3, G_a5
G_a2	74.12	4.23	G_a1, G_a3		74.13	4.14	G_a3		73.82	4.06	G_a3	
G_a3	78.44	4.15	G_a2		78.26	4.01	G_a2, G_a4		74.99	4.52	G_a2, G_a4	
G_a4	72.12	4.03	G_a5		71.87	3.96	G_a3		75.13	4.57	G_e1, G_a6	
G_a5	78.33	3.97	G_a4, G_a6		77.85	3.92	G_a4, G_a6		74.76	3.91	G_a6	
G_a6	62.62	4.35, 4.50	G_a5		62.46	4.47, 4.29	G_a5		61.65	4.35, 4.42	G_a5	
G_b1	106.03	4.86 (d, J = 7.5)	24, G_b2	G_b3, G_b2, G_b5	105.36	4.88 (d, J = 10.5)	24, G_b2	24, G_b3, G_b5	105.66	4.98 (d, J = 8.2)	24, G_b2	24, G_b2
G_b2	73.9	3.93	G_b1, G_b3		74.61	4	G_b3		73.24	4	G_b3	
G_b3	77.85	4.25	G_b2		76.59	3.92	G_b1, G_b2		78.34	4.4	G_b2, G_b3	
G_b4	71.58	4.12	G_b3, G_b5		80.93	4.29	G_d1, G_b3, G_b6		71.81	4.16	G_b3	
G_b5	75.25	3.9	G_b4		75.23	3.96	G_b6		77.56	4.06	G_b6	
G_b6	67.63	4.20, 4.51	G_b5, G_c1		62.76	4.51, 4.36	G_b5		61.82	4.35, 4.42	G_b5	
G_c1	99.44	5.44 (d, J = 3.0)	G_b6, G_c2	G_b6, G_c2								
G_c2	74.84	4.69	G_c1, G_c4									
G_c3	77.57	4.17	G_c4									
G_c4	72.18	4.13	G_c3, G_c5									
G_c5	75.25	4.19	G_c6									
G_c6	62.81	4.45	G_c5									
G_d1					102.8	5.88 (d, J = 2.8)	G_d2	G_b4, G_d2, G_d4				
G_d2					75.03	4.2	G_d1, G_d3					
G_d3					78.36	4.53	G_d2, G_d4					
G_d4					71.58	4.47	G_d3, G_d5					
G_d5					77.58	4. 03	G_d6					
G_d6					61.59	4.41, 4.37	G_d5					

Table S1. *Cont.*

No.	1				2				3			
	δ_C	δ_H	HMBC (C to H)	NOE [a] (H to H)	δ_C	δ_H	HMBC (C to H)	NOE [a] (H to H)	δ_C	δ_H	HMBC (C to H)	NOE [a] (H to H)
G_e1									90.48	5.90 (d, J = 3.5)	G_e2	G_a4, G_e2, G_e3
G_e2									81.35	4.22	G_f1, G_e1, G_e3	
G_e3									78.27	4.2	G_e2	
G_e4									71.51	4.19	G_e3, G_e6	
G_e5									74.82	4.05	G_e6	
G_e6									62.46 [b]	4.31, 4.50	G_e5	
G_f1									87.71	5.79 (d, J = 3.5)	G_f2	G_e2, G_f2, G_f3
G_f2									74.27	4.15	G_f1, G_f3, G_f4	
G_f3									76.38	4.31	G_f2, G_f4	
G_f4									71.67	4.18	G_f3, G_f5	
G_f5									75.2	4.03	G_f4, G_f6	
G_f6									62.48 [b]	4.31, 4.50	G_f5	

[a] Data from ROESY spectrum or NOE difference spectrum. [b] Interchangeable data.

Characterization Data of compound **4** and **5**.

Compound **4**: ESI-MS, *m/z* 1286 [M−1]$^{-}$; ^{13}C-NMR (Pyridine-D_5): δ 16.48, 18.50, 19.10, 23.19, 23.30, 24.02, 25.12, 25.40, 25.85, 26.81, 27.06, 28.15, 29.00, 33.40, 34.26, 35.79, 36.47, 39.50, 41.65, 42.87, 43.58, 46.93, 49.23, 50.26, 59.47, 60.52, 60.76, 68.54, 69.34, 71.27, 71.53, 71.82, 72.77, 72.94, 73.16, 73.36, 74.52, 74.61, 74.79, 76.22, 76.90, 77.03, 77.13, 77.39, 77.65, 77.75, 78.10, 78.23, 78.39, 87.34, 88.31, 99.70, 99.96, 100.28, 102.72, 104.22, 119.24, 144.01.

Compound **5**: ESI-MS, *m/z* 1286 [M−1]$^{-}$; ^{13}C-NMR (Pyridine-D_5): δ 16.79, 18.56, 19.06, 24.33, 24.48, 25.98, 26.51, 26.59, 27.42, 27.47, 28.03, 29.30, 29.57, 33.02, 34.31, 36.17, 36.60, 39.89, 40.86, 42.11, 43.28, 47.15, 49.46, 50.75, 61.56, 61.63, 62.53, 62.82, 67.81, 71.60, 71.71, 72.02, 72.08, 72.25, 72.39, 73.19, 73.46, 73.90, 74.31, 74.37, 74.61, 74.72, 74.87, 75.00, 75.24, 77.58, 77.85, 78.37, 78.43, 81.29, 82.10, 87.61, 92.11, 99.25, 102.78, 102.95, 106.07, 107.05, 118.24, 144.01.
